# PK/PD-Guided Strategies for Appropriate Antibiotic Use in the Era of Antimicrobial Resistance

**DOI:** 10.3390/antibiotics14010092

**Published:** 2025-01-14

**Authors:** Tetsushu Onita, Noriyuki Ishihara, Takahisa Yano

**Affiliations:** Department of Pharmacy, Shimane University Hospital, 89-1 Enya, Izumo 693-8501, Shimane, Japan

**Keywords:** pharmacokinetics, pharmacodynamics, antimicrobial resistance, population modeling, therapeutic drug monitoring, dose optimization

## Abstract

Antimicrobial resistance (AMR) poses a critical global health threat, necessitating the optimal use of existing antibiotics. Pharmacokinetic/pharmacodynamic (PK/PD) principles provide a scientific framework for optimizing antimicrobial therapy, particularly to respond to evolving resistance patterns. This review examines PK/PD strategies for antimicrobial dosing optimization, focusing on three key aspects. First, we discuss the importance of drug concentration management for enhancing efficacy while preventing toxicity, considering various patient populations, including pediatric and elderly patients with their unique physiological characteristics. Second, we analyze different PK modeling approaches: the classic top-down approach exemplified by population PK analysis, the bottom-up approach represented by physiologically based PK modeling, and hybrid models combining both approaches for enhanced predictive performance. Third, we explore clinical applications, including nomogram-based dosing strategies, Bayesian estimation, and emerging artificial intelligence applications, for real-time dose optimization. Critical challenges in implementing PK/PD simulation are addressed, particularly the selection of appropriate PK models, the optimization of PK/PD indices, and considerations concerning antimicrobial concentrations at infection sites. Understanding these principles and challenges is crucial for optimizing antimicrobial therapy and combating AMR through improved dosing strategies.

## 1. Introduction

Antimicrobial resistance (AMR) is one of the most serious global health threats of the 21st century. If no measures are taken, the death toll worldwide is estimated to reach 10 million by 2050, exceeding the number of cancer deaths [1]. The global significance of AMR has led to its recognition as a top health priority among the G7 nations, with the World Health Organization identifying AMR containment as a critical policy initiative requiring coordinated international action [2]. The emergence of AMR strains, including methicillin-resistant *Staphylococcus aureus* (MRSA), vancomycin-resistant enterococci, and multidrug-resistant *Pseudomonas aeruginosa*, poses significant clinical challenges because treatment options are currently severely limited.

To combat increasing AMR, optimizing the use of existing antibiotics through appropriate prescribing practices is crucial for maximizing therapeutic effectiveness while minimizing the development of resistance. The integration of pharmacokinetic (PK) and pharmacodynamic (PD) principles provides a scientific framework for optimizing antibiotic dosing regimens.

This review examines the critical aspects of PK/PD-guided antimicrobial therapy. First, we discuss the importance of drug concentration management, including PK/PD indices and their variations in special populations. Next, we explore population PK models and physiologically based PK models for the precise prediction of antibiotic exposure. We then address the clinical implementation of PK/PD simulations, including the application of artificial intelligence and machine learning approaches. Finally, we discuss key considerations and necessary precautions for the successful clinical application of these strategies.

## 2. Importance of Drug Concentration Management

Drug concentration management has a pivotal role in antimicrobial therapy from three critical perspectives: therapeutic efficacy, host factors, and toxicity prevention. This section explores these aspects in detail.

### 2.1. Enhancing Efficacy

Antimicrobial therapeutic effectiveness against bacterial infections is determined by the relationship between drug exposure parameters and bacterial susceptibility indices [3]. Drug exposure is characterized by key pharmacokinetic metrics, including the maximum drug concentration (C_max_), area under the concentration–time curve (AUC), and duration that drug concentrations exceed specific thresholds. These parameters were evaluated in relation to the minimum inhibitory concentration (MIC) of the target pathogen. The integration of pharmacokinetic and pharmacodynamic (PK/PD) parameters is fundamental to determining the optimal dosing strategies. Key PK/PD indices include the maximum concentration to MIC ratio (C_max_/MIC), the 24 h area under the curve to the MIC ratio (AUC_24_/MIC), and the percentage of time concentrations are above the MIC (%T > MIC). These parameters vary among antimicrobial classes and serve as critical indicators of therapeutic efficacy (Table 1) [4]. In empiric dosing decisions, the probability of target attainment (PTA) is a crucial consideration, as it predicts the likelihood of achieving therapeutic PK/PD targets in a population. A higher PTA can be achieved by increasing the dosage, adjusting the administration frequency, or implementing extended infusion. This probability-based approach helps optimize empiric dosing regimens, particularly when dealing with varying patient populations and potential pathogen susceptibilities.

**Figure 1:**
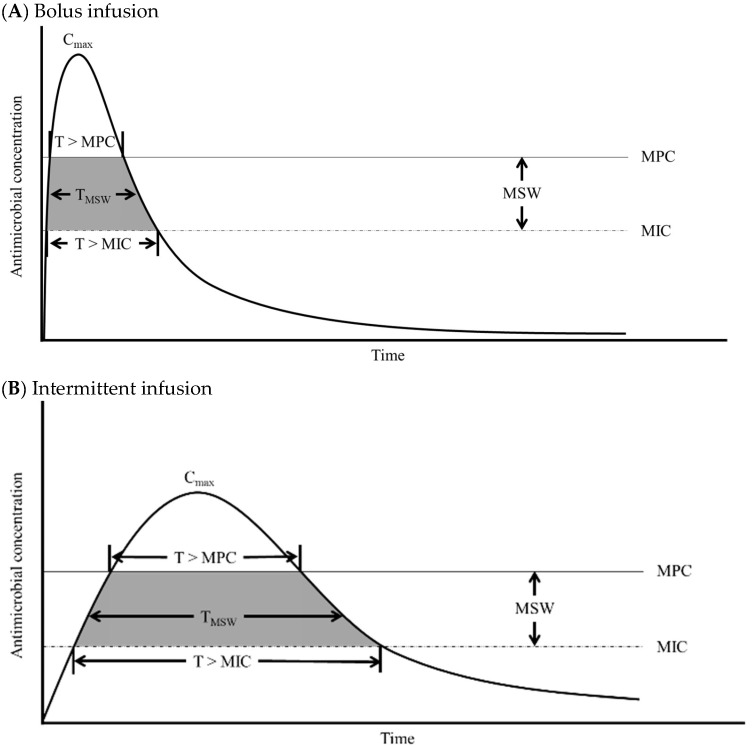

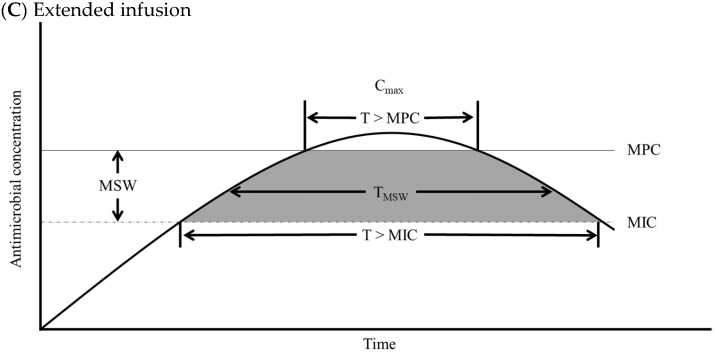
Schematic representation of key pharmacokinetic and pharmacodynamic (PK/PD) parameters in relation to antimicrobial resistance prevention: (**A**) bolus infusion, (**B**) intermittent infusion, and (**C**) extended infusion.

Beyond traditional MIC considerations, the mutant prevention concentration (MPC) is an important threshold for preventing the emergence of resistant bacterial strains. The concentration range between the MIC and MPC defines the mutant selection window (MSW), the zone in which resistant bacterial mutants are most likely to be selected (Figure 1). Precise monitoring of both the MIC and MPC is essential for optimizing antimicrobial regimens, particularly in cases involving resistant organisms for which higher doses or alternative antibiotics may be necessary [13]. In managing AMR infections, the focus often shifts from MIC-based to MPC-based indices to ensure effective therapeutic outcomes.

The diagram illustrates the relationship between antimicrobial concentration–time profiles and critical parameters including minimum inhibitory concentration (MIC) and mutant prevention concentration (MPC) for three administration methods: (A) bolus infusion, (B) intermittent infusion, and (C) extended infusion [13]. The most applied PK/PD indices are the area under the curve ratio (AUC/MIC, AUC/MPC), peak concentration ratio (C_max_/MIC, C_max_/MPC), and the percentage of time above the threshold (%T > MIC, %T > MPC). The mutant selection window (MSW) represents the concentration range between the MIC and MPC, and the periods when the antimicrobial concentration lies within the MSW are shown in the shaded area (TMSW).

### 2.2. PK Variations Based on the Host Factors

Host-specific characteristics significantly influence the PK of the antimicrobial agents. Special patient populations, including pediatric patients [14], elderly patients [15], patients with obesity [16], and patients with organ dysfunction (particularly renal and hepatic), exhibit distinct PK profiles that require careful consideration. In addition, changes in immune status [17] and various clinical conditions [18,19,20,21,22] have been reported to affect PK. The key PK variations to guide clinical decision-making across different patient groups is shown in Table 2.

#### 2.2.1. Pediatric Patients

PK/PD studies in pediatric patients are notably less extensive than those in adult studies. This population represents a particularly challenging group for drug-level management, as minimizing unnecessary drug exposure is crucial. Several approaches are available for PK/PD studies in children. Population pharmacokinetic analysis has been traditionally used to characterize drug disposition in children [23,24]. However, ethical issues often limit the ability to conduct large-scale studies. To overcome this limitation, meta-analyses of population pharmacokinetics have been employed, integrating data from case reports and previous studies to analyze drug concentrations and patient characteristics [25,26]. Additionally, physiologically based modeling approaches are increasingly being utilized to enhance our understanding of drug disposition in pediatric populations [27,28].

Studies are needed to determine safe and effective dosing, especially in neonates, with gestational ages ranging from 22 to 42 weeks, birth weights from 500 to 5000 g, and varying postnatal ages [29]. In neonates, vancomycin, a hydrophilic drug, may not have sufficient trough concentrations after the first empirical dose [30]. This is because neonates have a high body water content, which increases the distribution volume of the drug. Similarly, increases in the volume of distribution have been reported for lipophilic drugs such as fluoroquinolones, rifampicin, and linezolid [31]. This is likely attributable to the larger lipid-rich organs relative to body weight. For example, the absorption of ampicillin may be enhanced in neonates and infants due to immature gastric acid secretion [32]. However, it remains unclear whether this phenomenon is specific to penicillin antibiotics or a common characteristic of most antibiotics.

Drug clearance is primarily renal and hepatic clearance, which matures over time after birth, with relatively lower clearance values than in adults. Factors such as gestational length and calendar age require careful consideration in determining antimicrobial doses for neonates [33].

#### 2.2.2. Elderly Patients

In elderly patients, alterations in PK parameters, including absorption, distribution, metabolism, and excretion, can lead to prolonged drug half-life, increased drug toxicity, and an elevated risk of adverse reactions [34,35]. Although age-related decline in renal [36] and hepatic function [37] is common, the rate and extent of these changes vary significantly among patients [15]. Understanding both the physiological changes associated with aging and pathology-related alterations is crucial for appropriate drug dosing in this patient population.

Age-related changes in drug clearance are well documented through population PK studies. For example, piperacillin clearance shows significant age-related differences in Japanese populations: healthy young adults (mean age 25.1 years) demonstrated clearance of 11.9 L/h [38], while patients with community-acquired pneumonia (mean age 63.9 years) showed reduced clearance of 8.7 L/h [39]. This reduction was even more pronounced in elderly pneumonia patients over 75 years (mean age 86.5 years), with clearance of only 4.6 L/h [40]. These models incorporated creatinine clearance as a key covariate, which accounts for both age and weight effects. Pneumonia patients have been shown to have considerably reduced drug clearance. In addition, renal impairment occurred more frequently in elderly patients, although the causal relationship with drug clearance has not been fully elucidated. Careful attention should be paid to the design of dosing in elderly patients [41].

#### 2.2.3. Obesity and PK Alterations

Obesity is defined by the WHO as a body mass index (BMI) of 25 or greater, and in 2022, approximately 16% of adults worldwide aged 18 years and older were reported to be obese [42]. Changes in physiology due to obesity can have a significant impact on antimicrobial pharmacokinetics and may require changes in dosing regimens [43]. The first edition and the updated 2022 guideline provide specific dosage recommendations, including “no change to dose” for antimicrobials in obese patients [16,43]. For levofloxacin, a fluoroquinolone that is more fat-soluble than beta-lactams, a daily dose of 750 mg is recommended for Gram-negative bacterial infections in morbidly obese patients [44]. However, there is still insufficient information available, and some drugs are listed as having “Insufficient data” or “No data” in the guidelines [16].

#### 2.2.4. PK Changes in Organ Dysfunction and Clinical Conditions

Organ dysfunction substantially impacts drug disposition. Population PK analyses have shown that renal function, typically measured by creatinine clearance, is a significant covariate for drugs with renal elimination. For meropenem, the 2021 eGFR_cr-cysC_ equation, based on creatinine and cystatin C, has been shown to best predict meropenem clearance among various estimating equations for calculating the glomerular filtration rate [45].

Similarly, the presence or absence of hepatic dysfunction has been identified as an important covariate for drugs undergoing hepatic metabolism. For hepatically eliminated drugs, models have been reported that incorporate mild and moderate Child–Pugh classification as a covariate for clearance [46,47]. Various clinical conditions can also significantly affect drug disposition. Critical illness or sepsis can modify PK through alterations in organ perfusion, protein binding, and distribution volume. Additionally, altered immune status can affect drug metabolism through changes in inflammatory mediators. Models incorporating these physiological changes as covariates are still limited but are increasingly being developed for optimizing antimicrobial therapy in these complex patient populations.

### 2.3. Prevention of Antimicrobial Toxicity

The monitoring of blood antimicrobial concentrations can help prevent toxicity. The relationship between blood antimicrobial concentrations and toxicity has been well documented for various antibiotics. Specific adverse effects include vancomycin-induced nephrotoxicity [48,49], aminoglycoside-induced nephrotoxicity [50,51], linezolid-induced bone marrow suppression (including thrombocytopenia) [52,53,54], and cefepime-induced neurotoxicity [55,56,57]. Although these toxicities are generally reversible in most cases, appropriate antimicrobial concentration management can minimize the risk of adverse effects. Therapeutic drug monitoring (TDM) has been shown to effectively prevent drug-related toxicity [50,58,59,60].

## 3. PK Modeling for Predicting Antimicrobial Concentrations

Two major approaches (“top-down” and “bottom-up”) are predominantly used in constructing PK models for antimicrobial agents. The top-down approach represents a classic methodology, exemplified by population PK analysis, that develops PK models on the basis of observed clinical data [61]. This approach has been widely applied in clinical PK modeling to optimize antimicrobial dosing regimens. Recent studies have successfully used this approach to develop PK models for broad-spectrum antibiotics against drug-resistant bacteria and conduct PK/PD evaluations [40,62]. However, the top-down approach, including population PK analysis, requires substantial sampling, increasing both costs and ethical concerns.

The bottom-up approach, represented by physiologically based PK (PBPK) modeling, offers a more contemporary methodology based on a comprehensive understanding of human physiology and its mechanisms. PBPK models incorporate physiologically structured compartments reflecting the actual anatomical characteristics of organs and tissues [63,64]. A key advantage of PBPK modeling is its ability to predict PK with minimal sampling by incorporating existing data on organ blood flow, organ volume, and tissue-to-blood distribution ratios. This capability enables PK modeling in special populations, such as pediatric patients [65,66], and in complex scenarios including drug–drug interactions [67]. Numerous studies have used PBPK modeling to predict antimicrobial concentrations [68,69,70,71,72,73,74,75,76,77,78,79,80,81,82,83,84,85,86,87,88,89,90,91,92] (Table 3). However, model validation remains challenging, particularly in special populations where sampling is limited.

In addition, hybrid models combine physiological parameters, such as organ blood flow and volume (bottom-up approach), with conventional PK modeling (top-down approach). In antimicrobial research, these models have been particularly valuable for analyzing PK at infection sites with high pathogen concentrations, such as in the cerebrospinal fluid, prostate, and lung [93,94,95,96,97,98,99]. Although hybrid models effectively predict PK at various target sites by integrating previously published population PK parameters and tissue-to-blood distribution ratios, validation challenges persist due to the difficulty of target-organ sampling.

## 4. Clinical Applications Based on PK/PD Simulations

The success of antimicrobial therapy critically depends on achieving optimal drug concentrations in individual patients. This requires a precise understanding of patient-specific PK. This section introduces Model-Informed Precision Dosing, an approach based on various modeling strategies.

### 4.1. Dosing Strategies Based on Antimicrobial Nomograms

Nomograms are visual calculation tools that represent mathematical relationships through graphical parameters (covariates) and are used for dose calculations, risk assessments, and drug prognostication in clinical settings. These tools offer the advantage of rapid dose determination without requiring complex calculations or specialized software.

Several nomograms have been developed for various antimicrobial agents and clinical scenarios. For vancomycin, nomograms have been designed for specific patient populations, including obese adults [100], infants [101], patients with renal dysfunction [102,103], and patients with cirrhosis (where serum creatinine levels may be falsely low) [104]. Additional nomograms target specific therapeutic goals, such as achieving an AUC_0-24_ of 400 μg·h/mL [9,105] or predicting the risk of vancomycin-associated acute kidney injury in critically ill patients [106].

For β-lactam antibiotics, nomograms have been developed for meropenem in severe pneumonia [107], amoxicillin and cefazolin in infective endocarditis [108,109], and high-dose ceftriaxone in bacterial meningitis [110]. For aminoglycosides, studies have reported varying degrees of nomogram effectiveness: although beneficial for gentamicin dosing [111] and tobramycin [112], they have shown limited utility for amikacin in critically ill patients [113] and neonates [114]. In settings with limited resources, several implementation barriers exist. While therapeutic drug monitoring through measured drug concentrations is optimal, it may not be feasible in all healthcare environments due to cost, equipment, or expertise limitations. In such cases, practical alternatives include the use of validated nomograms, standardized dosing protocols based on population PK data, and simplified monitoring strategies. These approaches, while not ideal, can provide reasonable guidance for antimicrobial dosing when resources are constrained.

However, nomograms have important limitations. As they are developed for specific patient populations (e.g., obese, critically ill), they may provide inaccurate recommendations when applied to patients with different characteristics. Furthermore, they assume stable patient conditions and might not adequately account for dynamic changes during treatment. Therefore, careful consideration of the target patient population characteristics is essential when implementing nomogram-based dosing strategies.

### 4.2. Antimicrobial Dose Optimization Based on Bayesian Estimation

Bayesian estimation is widely used in TDM to estimate individual PK parameters. This approach combines population PK parameters (prior distribution) with observed drug concentrations (posterior distribution) to predict individual patient drug concentrations. In vancomycin therapy, Bayesian algorithm-guided dosing has demonstrated advantages over standard dosing, including reduced nephrotoxicity and faster improvement in inflammatory markers, such as C-reactive protein and procalcitonin [115]. The superior predictive performance of Bayesian methods has been well documented [116].

Recent guidelines for severe MRSA infections recommend using AUC-based monitoring for vancomycin, which requires at least two concentration measurements for accurate AUC determination [117,118]. However, the precision of the Bayesian estimation, similar to nomograms, depends heavily on the alignment between the population PK model and patient characteristics [119,120].

For β-lactam antibiotics, routine concentration-based dose optimization remains limited [121,122]. Although randomized controlled trials have evaluated the utility of TDM for specific scenarios, such as piperacillin and meropenem in critically ill patients [123] and piperacillin in patients with febrile neutropenia [124], such studies remain scarce. Alternative approaches include software-based dosing guidance using population PK parameters rather than individual Bayesian estimates, as demonstrated with meropenem [125,126].

### 4.3. Antimicrobial Dose Optimization Using Artificial Intelligence and Machine Learning

Recent years have witnessed the rapid advancement of artificial intelligence (AI) and machine learning applications across various medical fields. In antimicrobial dose optimization, although these approaches are still emerging, several notable applications have been reported.

The majority of studies have focused on glycopeptide antibiotics, particularly vancomycin, for which initial dosing algorithms [127,128,129,130,131] and blood concentration prediction models have been developed. Similar approaches have been reported for teicoplanin [132]. The extensive clinical data available for vancomycin, accumulated through decades of recommended therapeutic drug monitoring [133], makes it particularly suitable for AI model development.

AI-based approaches are particularly promising in handling complex patient populations, as they can effectively integrate and analyze large-scale clinical data to enhance dose optimization strategies. These approaches are especially valuable when extensive therapeutic drug monitoring data are available, enabling more precise dosing predictions. However, AI/ML approaches in antimicrobial dosing face several important limitations. First, prediction accuracy heavily depends on dataset quality, including issues such as data completeness, potential bias in patient selection, and the standardization of measurements. Second, the ’black box’ nature of many AI algorithms makes it difficult to understand and validate the decision-making process, which is crucial in clinical applications. Third, validation across different clinical settings remains challenging, particularly for special populations where data are limited. These limitations necessitate the careful consideration of the underlying datasets and validation methods when implementing AI-based approaches in clinical practice.

## 5. Challenges in the Clinical Implementation of PK/PD Simulation

### 5.1. Appropriate Selection of PK Models

The successful implementation of PK/PD simulation requires the careful selection of PK models that align with drug characteristics and patient pathophysiology. This is particularly crucial for antimicrobial agents, which are predominantly water-soluble and renally eliminated, making renal function a critical consideration in model selection.

Vancomycin can serve as an illustrative example, with numerous PK models developed for specific patient populations, including those with renal dysfunction [69,134], augmented renal clearance [135,136,137], and patients undergoing renal replacement therapy [138,139,140]. In populations in which creatinine-based renal function estimates may be unreliable (e.g., elderly patients and patients with muscular dystrophy), PK models incorporating cystatin C-based estimations may offer improved accuracy [119,141]. Additional specialized models have been developed for specific patient populations, including pediatric patients [142], burn victims [143], and individuals with febrile neutropenia [144]. These models reflect the importance of considering pathophysiological factors beyond renal function.

Model selection should prioritize alignment between patient characteristics and the population used to develop the PK model. Key considerations include not only demographic factors, such as age and weight, but also biomarkers reflecting organ function, particularly those involved in drug elimination.

### 5.2. Optimization of the Antimicrobial PK/PD Indices

Selecting appropriate PK/PD indices is crucial for predicting antimicrobial efficacy. Although various indices exist, selection should be based on the specific bactericidal characteristics of each antimicrobial class (e.g., %T > MIC for β-lactams). Target values for these indices vary significantly in the literature. For example, with penicillins, %T > MIC targets of 30% for bacteriostatic effect and 50% for bactericidal effect have been reported [8]. The choice of target significantly affects dosing recommendations, with inadequate targets risking therapeutic failure and excessive targets potentially increasing adverse effects. For severe infections, higher targets, such as 100% *f*T > MIC, may be warranted [145], emphasizing the need for patient-specific PK/PD target selection.

The MIC determination methodology represents another critical consideration. Various methods exist, including broth microdilution and the E-test, each potentially yielding different results. Rosa et al. demonstrated only 33% concordance between the broth microdilution and E-test methods for vancomycin susceptibility testing against *Staphylococcus aureus* [146]. Similar discrepancies have been reported by Kays et al. for newer fluoroquinolones against *S. pneumoniae* [147]. Underestimation of true MIC values can cause artificially elevated PK/PD indices, potentially leading to suboptimal dosing.

### 5.3. Considerations for Antimicrobial Concentrations at Infection Sites

Most PK/PD-based dosing optimization studies use plasma or serum drug concentrations due to sampling accessibility. However, this approach might not accurately reflect drug concentrations at the actual infection sites where pathogenic bacteria are present (e.g., cerebrospinal fluid in meningitis or prostate tissue in prostatitis). Although blood concentrations are commonly measured, infection-site concentrations are often more clinically relevant, although their PK/PD relationships remain inadequately characterized.

Antimicrobial concentrations at infection sites may be substantially lower than plasma or serum concentrations due to the free-drug tissue distribution [148], potentially causing the overestimation of therapeutic efficacy based solely on blood concentrations. This consideration is particularly crucial for antimicrobials with limited tissue penetration. Site-specific PK/PD studies have been conducted to optimize dosing regimens for severe infections, such as meningitis, pneumonia, and prostatitis [97,98,99,149].

## 6. Conclusions

The PK/PD-based optimization of antimicrobial therapy is crucial for controlling AMR and improving clinical outcomes, especially against resistant pathogens. Achieving this goal requires a detailed understanding of drug-specific PK/PD targets and patient-related factors, as well as the integration of advanced modeling techniques, such as PBPK, Bayesian approaches, and AI-driven tools, into routine care. Future research should focus on validating PK/PD approaches through clinical outcome studies, expanding our understanding in special populations and evaluating new technologies for antimicrobial dosing optimization. These efforts will enhance our ability to provide more effective and personalized antimicrobial therapy while supporting sustainable AMR management.

## Figures and Tables

**Table 1:** Classification of antimicrobials based on PK/PD indices for optimal dosing strategies [4].

	Antimicrobial Activity	PK/PD Index	Target PK/PD Index
Concentration-dependent activity	Aminoglycosides	*f*C_max_/MIC *f*AUC_24_/MIC	C_max_/MIC > 8–10 [5]
	Quinolones		AUC_0-24_/MIC > 125 [6] C_max_/MIC > 10–12 [7]
Time-dependent activity	Penicillins Cephalosporins Carbapenems	*f*T > MIC	30, 50 * [8] 40, 60–70 * [8] 20, 40 * [8]
Concentration-dependent activity with time-dependence	Vancomycin Fosfomycin Linezolid Daptomycin	*f*AUC_24_/MIC	400 [9] 23 [10] 100 [11] 666 [12]

*f*C_max_/MIC: ratio of free maximum drug concentration to the minimum inhibitory concentration; *f*T > MIC: percentage of time that free drug concentrations exceed the minimum inhibitory concentration over 24 h; *f*AUC_24_/MIC: ratio of the free drug 24 h area under the concentration–time curve to the minimum inhibitory concentration *: First value represents bacteriostatic target and second value represents bactericidal target.

**Table 2:** Key considerations of PK variations in different patient groups.

Patient Group	Key Consideration
Pediatrics [14]	Body size Organ function (which is defined by age)
Elderly patients [15]	Age-related changes in organ mass Blood circulation alongside changes in body composition Disease-associated changes in the organs and systems functioning
Obesity [16]	Increased total body weight via increased adipose and muscle mass Increased organ mass

**Table 3:** PBPK models for predicting the antibiotic concentration.

	Blood Concentrations	Tissue or Fluid Concentrations
Adults	Ceftazidime [68,69]	Azithromycin [70] (lung epithelial-lining fluid)
	Cefuroxime [69,71]	Ciprofloxacin [72,73] (bone, etc.)
	Vancomycin [69,81]	Ertapenem [74,75] (kidney, fat, intestines, etc.)
		Levofloxacin [76] (muscle, liver, kidney, skin)
		Meropenem [77] (lung epithelial-lining fluid)
		Moxifloxacin [78] (muscle interstitium, fat interstitium)
		Oxytetracycline [79] (kidney, liver, and muscle)
		Solithromycin [80] (lung epithelial-lining fluid)
Pediatrics	Amikacin [82]	Ciprofloxacin [84] (urine, saliva)
	Ceftazidime [83]	
	Clindamycin [85]	
	Ertapenem [86]	
	Fosfomycin [87]	
	Linezolid [88]	
	Meropenem [87]	
	Moxifloxacin [89]	
	Trimethoprim Sulfamethoxazole [90]	
All age	Ciprofloxacin [91]	
	Gentamicin [92]	
	Tobramycin [92]	
	Vancomycin [92]	
